# Gridless DOA Estimator for 1.5-Bit Sparse Massive MIMO Systems Based on Covariance Matrix Estimation

**DOI:** 10.3390/e28060605

**Published:** 2026-05-28

**Authors:** Yuan Peng, Xiongbo Zheng, Zhiyong Cheng

**Affiliations:** 1 College of Mathematical Sciences, Harbin Engineering University, Harbin 150001, China; pengyuanedu@hrbeu.edu.cn; 2 CETC Taiji Computer Co., Ltd., Beijing 100102, China; 3 CETC Big Data Research Institute, Guiyang 550008, China; 4 School of Computer Science and Artificial Intelligence, Chaohu University, Hefei 238024, China; chengzhiyong@ieee.org

**Keywords:** 1.5-bit quantization, sparse array, direction-of-arrival (DOA) estimation, covariance matrix estimation

## Abstract

To reduce the hardware cost of massive multiple-input multiple-output (MIMO) systems, low-bit analog-to-digital converters (ADCs) and sparse arrays are widely used. Compared with traditional 1-bit and 2-bit quantization techniques, 1.5-bit quantization uses two symmetric non-zero thresholds to quantize signal power into three levels, thereby balancing quantization complexity against system performance. However, the quantization loss introduced by 1.5-bit quantization is still significant and leads to degradation in DOA estimation performance. To improve the DOA estimation accuracy of 1.5-bit sparse massive MIMO systems, a covariance matrix estimation method is proposed. This method exploits the Toeplitz property of the covariance matrix of sparse arrays and the relationship between 1.5-bit quantized signals and their unquantized counterparts to transform the covariance matrix estimation problem for 1.5-bit sparse arrays into a non-convex optimization problem with equality constraints. We then further exploit the properties of 1.5-bit quantized signals to relax this problem into a convex problem and solve it via semidefinite programming. Once the covariance is estimated, the DOAs can be recovered by subspace-based methods. Numerical results show that the proposed method achieves higher estimation accuracy than 1.5B-MUSIC and 1-bit covariance-fitting baselines on 1.5-bit sparse arrays, and is competitive with structured covariance-fitting baselines applied to unquantized data, especially on coprime arrays in low-snapshot scenarios.

## 1. Introduction

With the increasing amount of information transmitted in modern society, massive multiple-input multiple-output (MIMO) systems are widely used to meet the demand for high-data-rate transmission [1]. However, the use of a large number of antenna elements and high-resolution analog-to-digital converters (ADCs) [2] in massive MIMO systems leads to significant increases in hardware cost and power consumption. To mitigate the impact of growing hardware expense, sparse arrays [3] and low-bit ADCs have been proposed and widely adopted. Sparse arrays effectively enhance the degrees of freedom and resolution of an array by employing non-uniform element layouts with a limited number of antenna elements; common examples include coprime arrays and nested arrays. Low-bit ADCs, on the other hand, effectively reduce system complexity by decreasing the number of quantization bits per sampling point. However, the reduction in quantization bits and the increase in array complexity pose greater challenges for signal processing. Direction-of-arrival (DOA) estimation, a fundamental problem in array signal processing, is one of the key challenges that must be addressed.

A typical low-bit quantization technique is 1-bit quantization. This technique uses a comparator to quantize continuous-amplitude signals into 1-bit signals that contain only sign information, significantly reducing the hardware complexity and power consumption of ADCs. When the threshold of the comparator is set to zero, the scheme is referred to as zero-threshold 1-bit quantization, which has a long research history. The correlation coefficient estimation problem for zero-threshold 1-bit sampled signals was studied in [4], which introduced the well-known arcsine law. The authors of [5,6] presented maximum-likelihood-based 1-bit DOA estimation methods and provided performance analyses. The Cramér–Rao lower bound (CRLB) of zero-threshold 1-bit quantization under sparse arrays was analyzed in [7]. The authors of [8] extended the DOA estimation problem for 1-bit sparse arrays to polarization-sensitive arrays. Other applications of 1-bit quantization techniques can be found in [9,10,11,12]. To address the fact that zero-threshold 1-bit quantization cannot recover signal amplitude information, researchers have proposed biased 1-bit quantization techniques. The authors of [13] presented a fixed-threshold 1-bit autocorrelation estimation method, whose estimation performance was analyzed in [14]. Closely related, ref. [15] addressed the recovery of the full (i.e., diagonal-included) covariance matrix from non-zero-threshold 1-bit samples, deriving the Fisher information matrix and characterizing the dependence of the recovery MSE on the threshold; this work makes it clear that under 1-bit sampling the optimal threshold varies with the unknown variances and correlations, motivating the multi-level or time-varying schemes considered next. A time-varying-threshold 1-bit parameter estimation method was proposed in [16] and applied to MIMO radar. Building on the modified arcsine law, ref. [17] extended 1-bit covariance recovery to non-stationary signals with time-varying sampling thresholds, recovering both the time-varying variance and the autocorrelation values of the input. Both refs. [15,17] therefore tackle the same underlying difficulty—the loss of amplitude information under 1-bit sampling—through richer (non-zero or time-varying) thresholds, rather than through a finer quantization grid. However, whether zero-threshold or biased, 1-bit quantization techniques both introduce significant quantization errors, which lead to degraded DOA estimation performance. To address this, researchers have proposed multi-bit quantization techniques to improve DOA estimation performance. A MIMO parameter estimation method based on mixed-bit ADCs was proposed in [18], and ref. [19] presented a low-bit covariance matrix estimation method. However, increasing the number of bits also means further increasing hardware cost, which contradicts the original intent of low-bit quantization.

To strike a balance between performance and complexity, a 1.5-bit quantization technique was proposed in [20] and has shown strong application prospects. The 1.5-bit quantization technique quantizes signal amplitudes into three levels using two symmetric non-zero thresholds, thereby retaining more information than traditional 1-bit quantization. Compared with 2-bit quantization, 1.5-bit quantization uses one fewer comparator, which further reduces hardware complexity and power consumption. The authors of [21] subsequently provided a closed-form solution for covariance matrix recovery under 1.5-bit quantization and analyzed the DOA estimation performance obtained from this recovered covariance matrix. It should be noted that all theoretical results in [21] are based on infinite-snapshot scenarios, so their performance for DOA estimation in low-snapshot scenarios is unknown. In addition, for sparse arrays, the method in [20] requires constructing difference co-array outputs. However, difference co-arrays reduce the statistical efficiency of the received signals [22], which causes the DOA estimation accuracy at high signal-to-noise ratios (SNRs) to deviate from the CRLB.

Existing sparsity-based methods provide new tools to address the above two issues. For the processing of 1-bit quantized signals, refs. [23,24] presented signal reconstruction methods based on convex optimization and iterative hard thresholding, respectively, to recover the normalized signals prior to 1-bit compressed quantization; refs. [25,26] provided gridless sparse line-spectrum estimation methods for 1-bit quantization. The above methods transform the signal recovery or parameter estimation problem after 1-bit quantization into a convex optimization problem with sign constraints; this approach does not rely on the statistical characteristics of the signal and is independent of the number of snapshots. For the processing of sparse-array received signals, refs. [27,28] transformed the DOA estimation problem into nuclear-norm and atomic-norm optimization problems, thereby eliminating the dependence of DOA estimation on differential array outputs, while the Sparse and Parametric Approach (SPA) [29] and Enhanced Matrix Completion (EMaC) [30] exploited the structure of Toeplitz and Hankel matrices, respectively, to fit the covariance matrix of sparse arrays. The above methods treat the elements of a sparse array as sparse spatial-domain samples and use sparse reconstruction algorithms to reconstruct the covariance matrix of a uniform linear array with the same degrees of freedom, without the need to construct differential array outputs, while effectively suppressing finite-snapshot errors. By combining these two lines of work, the DOA estimation problem for 1.5-bit sparse arrays can be transformed into a sparsity-based reconstruction problem with sign constraints, which allows reconstruction of the normalized unquantized received-signal covariance matrix of a uniform linear array with the same degrees of freedom, effectively suppressing finite-snapshot errors and avoiding the loss of statistical efficiency caused by difference co-arrays.

Motivated by the above observations, this paper proposes a DOA estimation algorithm based on 1.5-bit sparse covariance matrix estimation (1.5B-SCE) for 1.5-bit sparse arrays. In contrast to the closed-form covariance recovery of [21], which is accurate only in the infinite-snapshot regime, and in contrast to the difference-coarray-based 1.5B-MUSIC of [20], which suffers from statistical-efficiency loss [22], 1.5B-SCE directly formulates the recovery of the unquantized ULA covariance as a constrained covariance-fitting problem. The novelty of this paper lies at the formulation level: the three ingredients used here—sign consistency of low-bit quantization, Toeplitz covariance fitting on sparse arrays, and Schur-complement relaxation—have each been studied individually in prior work, but to our knowledge they have not previously been combined into a single SDP tailored to 1.5-bit sparse-array DOA estimation. The resulting formulation (i) keeps the information in the three-level {−1,0,+1} output through a sign-product inequality, (ii) enforces the Toeplitz structure of the underlying ULA covariance without resorting to difference co-arrays, and (iii) remains convex and can therefore be solved to global optimality via standard SDP. We further note that the present work is complementary to, rather than a refinement of [15,17]: those works recover the covariance of 1-bit samples by exploiting non-zero or time-varying thresholds within the (modified) arcsine-law framework, whereas 1.5B-SCE works in the 1.5-bit (three-level {−1,0,+1}) regime on a sparse array, replaces the closed-form arcsine recovery by a sign-product inequality inside an explicitly convex SDP, and simultaneously enforces the Toeplitz structure of the underlying ULA covariance to suppress finite-snapshot errors. The gain over the closed-form 1.5-bit recovery of [21] therefore comes from the structured covariance-fitting regularization rather than from a tighter arcsine relation. Simulation experiments show that 1.5B-SCE effectively improves the DOA estimation accuracy on 1.5-bit sparse arrays compared with 1.5B-MUSIC, and that on coprime arrays in the low-snapshot regime its accuracy is competitive with structured covariance-fitting baselines applied to unquantized data. We note that 1.5B-SCE does not recover information that was lost during quantization; its advantage over plain MUSIC on unquantized sparse arrays comes from the structured covariance-fitting regularization, not from the quantization itself as our extended baselines in Section 4 make explicit.

The rest of this paper is organized as follows. Section 2 introduces the signal model of 1.5-bit sparse arrays; Section 3 presents the specific implementation steps of 1.5B-SCE; Section 4 validates the effectiveness of the proposed method through simulation experiments; and finally, Section 5 concludes the paper.

## 2. Signal Model

Consider *K* plane-wave signals arriving from the far field at a linear array composed of *M* elements, with the element positions determined by the set S, which we refer to as the array configuration set. The position of the *m*-th element is given by $\omega_{m}\lambda /2$, where $\omega_{m}\in S$ and λ is the signal wavelength. The received signal $x_{m}\left(t\right)$ at the *m*-th element is given by

(1) $$x_{m}\left(t\right)=\sum_{k=1}^{K}s_{k}\left(t\right)e^{j\pi \omega_{m}{\bar{\theta }}_{k}}+n_{m}\left(t\right),m=1,2,\ldots ,M$$

where $s_{k}\left(t\right)$ is the baseband complex amplitude of the *k*-th signal, ${\bar{\theta }}_{k}=\sin\left(\theta_{k}\right)$, $\theta_{k}$ is the DOA of the *k*-th signal, and $n_{m}\left(t\right)$ is additive complex Gaussian white noise at the *m*-th element, satisfying $n_{m}\left(t\right)∼\mathrm{CN}\left(0,\sigma^{2}\right)$. Stacking the received signals of all elements into a vector, we obtain

(2) $$x\left(t\right)=A\left(\bar{\theta }\right)s\left(t\right)+n\left(t\right)$$

where $x\left(t\right)={\left[x_{1}\left(t\right),\ldots ,x_{M}\left(t\right)\right]}^{T}$, $s\left(t\right)={\left[s_{1}\left(t\right),\ldots ,s_{K}\left(t\right)\right]}^{T}$, $n\left(t\right)={\left[n_{1}\left(t\right),\ldots ,n_{M}\left(t\right)\right]}^{T}$, $\bar{\theta }={\left[{\bar{\theta }}_{1}\ldots ,{\bar{\theta }}_{K}\right]}^{T}$, $A\left(\bar{\theta }\right)=\left[a\left({\bar{\theta }}_{1}\right),a\left({\bar{\theta }}_{2}\right),\ldots ,a\left({\bar{\theta }}_{K}\right)\right]$ is the array steering matrix, and $a\left({\bar{\theta }}_{k}\right)={\left[e^{j\pi \omega_{1}{\bar{\theta }}_{k}},e^{j\pi \omega_{2}{\bar{\theta }}_{k}},\ldots ,e^{j\pi \omega_{M}{\bar{\theta }}_{k}}\right]}^{T}$ is the array steering vector of the *k*-th signal.

In this paper, we make the following assumptions about the sources and noise: (1) all *K* sources are independent far-field Gaussian sources; (2) the DOAs of the different sources are distinct; (3) the noise at all elements follows independent and identically distributed complex Gaussian distributions; and (4) the noise is statistically independent of the sources.

Let us now consider the array configuration. When the array configuration set S consists of consecutive non-negative integers starting from 0, i.e., S={0,1,2,…,M−1} (so that S has exactly *M* elements, consistent with the assumption above), we refer to this array as a uniform linear array (ULA). When the elements of S are not consecutive, it is referred to as a sparse linear array (SLA). There are two common SLA configurations. The first is the nested array [31], which consists of a ULA with element spacing 1 and $M_{1}$ elements, nested with another ULA with element spacing $M_{1}$ and $M_{2}$ elements. Its array configuration set is given by

$$S_{n}=\left\{1,2,\ldots ,M_{1},M_{1}+1,\ldots ,M_{2}\left(M_{1}+1\right)\right\}.$$

The second type of sparse array is the coprime array [32], which consists of two ULAs with element spacings $M_{2}$ and $M_{1}$ and with $M_{2}$ and $2M_{1}-1$ elements, respectively, where $M_{1}$ and $M_{2}$ are coprime. Its array configuration set is given by

$$S_{c}=\left\{0,M_{1},\ldots ,M_{2}M_{1},M_{2},\ldots ,2\left(M_{1}-1\right)M_{2}\right\}.$$

Other sparse arrays include minimum redundancy arrays [33], super nested arrays [34], and least hole arrays [35]. For simplicity, in the sequel we refer to an array with configuration set S simply as S. For example, a coprime array with configuration set $S_{c}$ is referred to as $S_{c}$.

This paper considers the 1.5-bit quantization technique, which quantizes the received signal into three levels using two symmetric non-zero thresholds ±λ. The quantization is defined as follows:

(3) $$Q\left(t\right)=\left\{\begin{array}{cc} 1, & t>\lambda \\ 0, & |t|\leq \lambda \\ -1, & t<-\lambda \end{array}\right.,$$

where Q(·) is the quantization function, and λ is a non-negative threshold. For complex signals, 1.5-bit quantization is performed separately on the real and imaginary parts, resulting in the quantized signal:

(4) $$y_{m}\left(t\right)=Q\left(ℜ\left(x_{m}\left(t\right)\right)\right)+jQ\left(ℑ\left(x_{m}\left(t\right)\right)\right),m=1,2,\ldots ,M$$

where ℜ(·) and ℑ(·) denote the real and imaginary parts, respectively. Stacking the quantized signals of all elements into a vector, we have

(5) y(t)=Q(ℜ(x(t)))+jQ(ℑ(x(t)))

Assuming that the array received signals are quantized with the 1.5-bit quantizer and that *N* snapshots are collected, the resulting set of quantized signal samples is ${\left\{y\left(t_{n}\right)\right\}}_{n=1}^{N}$. The sample covariance matrix of the quantized signals can be estimated as

(6) $$R_{y}=\frac{1}{N}\sum_{n=1}^{N}y\left(t_{n}\right)y^{H}\left(t_{n}\right)$$

where ${\left(\cdot \right)}^{H}$ denotes the conjugate transpose operation.

## 3. Proposed Method

In this section, we propose a DOA estimation algorithm based on 1.5-bit sparse covariance matrix estimation (1.5B-SCE) that estimates the signal DOAs from the sample covariance matrix in (6). In ref. [21], the authors provided a closed-form solution for covariance matrix recovery under 1.5-bit quantization in the infinite-snapshot regime. However, this closed-form solution is applicable only in the infinite-snapshot regime; in the finite-snapshot regime the recovered covariance matrix has significant errors, which lead to degraded DOA estimation performance. Moreover, the method in [20] requires constructing difference co-array outputs, which reduces the statistical efficiency of the received signals [22] and causes the DOA estimation accuracy at high SNR to deviate from the CRLB.

Before presenting the derivation, we summarize the notation used in this section in Table 1 so that the role of each matrix (observed, modeling target, optimization variable, or derived auxiliary) is unambiguous.

The core problem of DOA estimation under low-snapshot sampling is to recover the covariance matrix of the unquantized received signals, that is, to estimate the unquantized received-signal covariance matrix $R_{S}=E\left\{x\left(t\right)x^{H}\left(t\right)\right\}$ from the quantized covariance matrix in (6). For sparse arrays, it is also necessary to reconstruct the covariance matrix $R_{D}$ of a uniform linear array D with the same degrees of freedom from the estimated $R_{S}$.

We express the signal model in (2) in matrix form as

(7) $$X_{S}=A_{S}\left(\bar{\theta }\right)S+N_{S}$$

where $X_{S}\in C^{M\times N}$, $S\in C^{K\times N}$, and $N_{S}\in C^{M\times N}$ collect the unquantized snapshots, source-signal vectors, and noise vectors columnwise, i.e., $X_{S}=\left[x\left(t_{1}\right),\ldots ,x\left(t_{N}\right)\right]$, $S=[s\left(t_{1}\right),\ldots ,s\left(t_{N}\right)]$, and $N_{S}=\left[n\left(t_{1}\right),\ldots ,n\left(t_{N}\right)\right]$, and $A_{S}\left(\bar{\theta }\right)\in C^{M\times K}$ is the array steering matrix; the source-matrix symbol S (bold–italic) is distinct from the array configuration set S (blackboard bold). The relationship between the received-signal matrix of array D and $X_{S}$ can be expressed as

(8) $$X_{S}=\Gamma X_{D}$$

where Γ is the selection matrix from array D to array S. Here, the virtual ULA $D=\{0,1,\ldots ,\omega_{M}\}$ is the smallest ULA whose aperture matches that of S; we denote its size by $M_{D}=\omega_{M}+1$. The selection matrix $\Gamma \in {\left\{0,1\right\}}^{M\times M_{D}}$ has a single 1 in row *m* at column $\omega_{m}+1$ (and zeros elsewhere) so that $\Gamma X_{D}$ retains exactly the rows of $X_{D}$ indexed by S.

The sample covariance matrix ${\hat{R}}_{D}$ of D is then expressed as

(9) $${\hat{R}}_{D}=\frac{1}{N}X_{D}X_{D}^{H}.$$

Since ${\hat{R}}_{D}$ is an unbiased estimate of $R_{D}$, we have

(10) $$E\left\{\mathrm{vec}\left({\hat{R}}_{D}\right)\right\}=\mathrm{vec}\left(R_{D}\right),$$

where vec(·) denotes the matrix vectorization operator, and E{·} denotes the expectation operator. The process of using generalized least squares to solve for $R_{D}$ can be expressed as

(11) $$\begin{array}{c} \min_{R_{D}}\frac{1}{\omega_{M}}\left(\mathrm{vec}\left({\hat{R}}_{D}\right)-\mathrm{vec}\left(R_{D}\right)\right)(R_{D}^{-T}⊗\\ R_{D}^{-1})\left(\mathrm{vec}\left({\hat{R}}_{D}\right)-\mathrm{vec}\left(R_{D}\right)\right), \end{array}$$

where ⊗ denotes the Kronecker product. Equation (11) can be further simplified, as in [29], to

(12) $$\min_{R_{D}}{\left\|R_{D}^{-\frac{1}{2}}\left({\hat{R}}_{D}-R_{D}\right)R_{D}^{-\frac{1}{2}}\right\|}_{F}^{2},$$

where the subscript *F* denotes the Frobenius norm of a matrix, also referred to as the *F*-norm. As shown theoretically in [36], (12) yields the maximum-likelihood solution of $R_{D}$. However, (12) is non-convex, so its global optimum cannot be obtained with standard convex optimization algorithms. To address this, we adopt the convex approximation of [37] to transform it into a convex problem. The convex approximation of (12) is

(13) $$\min_{R_{D}}{\left\|R_{D}^{-\frac{1}{2}}\left({\hat{R}}_{D}-R_{D}\right)\right\|}_{F}^{2}.$$

The covariance matrix fitting above is only for unquantized received signals. For signals after 1.5-bit quantization, the key to applying covariance-matrix-fitting techniques to 1.5-bit quantized sparse arrays is how to combine the characteristics of 1.5-bit quantization with covariance matrix fitting. Since (13) obtains the estimate $R_{D}$ from the value of ${\hat{R}}_{D}$, we must first establish the relationship between the 1.5-bit quantized signal y(t) and ${\hat{R}}_{D}$. If this relationship is used as a constraint, a constrained optimization problem can be formulated based on (13) to recover the covariance matrix of the unquantized received signals from 1.5-bit quantized data.

Let the matrix form of the received signal y(t) of array S be $Y_{S}$. According to the definition of 1.5-bit quantization, with quantization threshold λ we have

(14) $$Y_{S}=Q\left(ℜ\left(X_{S}\right)\right)+jQ\left(ℑ\left(X_{S}\right)\right),$$

and

(15) $$Q\left(x\right)=\frac{1}{2}\left(\mathrm{sign}\left(x-\lambda \right)+\mathrm{sign}\left(x+\lambda \right)\right),$$

where sign(·) is the sign function. Equation (15) is equivalent to the definition in (3) as shown by the following lemma.

**Lemma** **1.** *The 1.5-bit quantization in (3) can equivalently be expressed as*

(16) $$Q\left(x\right)=\frac{1}{2}\left(\mathrm{sign}\left(x-\lambda \right)+\mathrm{sign}\left(x+\lambda \right)\right).$$

**Proof.**  Since λ is symmetric about 0, we only consider the case λ≥0.

If λ=0, the results in (3) and (15) both degenerate to traditional 1-bit quantization. If λ>0, then for any x<−λ we have Q(x)=−1, sign(x−λ)=sign(x+λ)=−1, and $\frac{1}{2}\left(\mathrm{sign}\left(x-\lambda \right)+\mathrm{sign}\left(x+\lambda \right)\right)=-1$, so the results in (3) and (15) are the same. The cases x>λ and |x|≤λ can be proved analogously.    □

The relationship in (14) and (15) is referred to as the sign consistency between the 1.5-bit quantized signal $Y_{S}$ and the unquantized received signal $X_{S}$. We further simplify (14) as

(17) $$Y_{S}=Q\left(X_{S}\right).$$

The relationship between $X_{S}$ and ${\hat{R}}_{D}$ is determined by (8) and (9). By incorporating (8), (9), and (17) as constraints in the covariance-matrix-fitting optimization problem, we obtain

(18) $$\begin{array}{l} \min_{R_{D}}\;\|R_{D}^{-\frac{1}{2}}\left({\hat{R}}_{D}-R_{D}\right){\|}_{F}^{2},\\ \;\;s.t.X_{S}=\Gamma X_{D},\\ \;\;\;\;\;\;\;\;\;{\hat{R}}_{D}=\frac{1}{N}X_{D}X_{D}^{H},\\ \;\;\;\;\;\;\;\;\;Y_{S}=Q\left(X_{S}\right). \end{array}$$

Clearly, the three constraints in (18) are all equality constraints, which makes (18) non-convex and not directly solvable by convex optimization algorithms. However, by using convex relaxation, (18) can be transformed into a convex optimization problem and solved via semidefinite programming.

**Relaxation roadmap.** Before presenting the derivation, we classify the three transformations that will be applied so that their status—equivalent reformulation versus genuine relaxation—is unambiguous. (i) The Frobenius objective in (18) is rewritten as a linear objective plus a Schur-complement LMI by introducing an auxiliary variable W; this step is an equivalent reformulation and does not enlarge the feasible set. (ii) The sample-covariance equality ${\hat{R}}_{S}=\frac{1}{N}X_{S}X_{S}^{H}$ is replaced by the PSD inequality $N{\hat{R}}_{S}⪰X_{S}X_{S}^{H}$; this is the standard Schur-complement relaxation used in atomic-norm/SPA-style covariance fitting and introduces a small outward relaxation of the feasible set. (iii) The sign-consistency equality $Y_{S}=Q\left(X_{S}\right)$ is replaced by a sign-product inequality between $X_{S}$ and $Y_{S}$; this is the only genuinely loose relaxation in our formulation, and the quantitative effect of losing the quantization-threshold information in the forward direction is discussed in the remark following (32).

First, consider the minimization term in (18). Using properties of the matrix *F*-norm, we have

(19) $$\begin{array}{l} \min_{R_{D}}\;\|R_{D}^{-\frac{1}{2}}\left({\hat{R}}_{D}-R_{D}\right){\|}_{F}^{2}\\ \;\;\;=\min_{R_{D}}\mathrm{Tr}\left(\left({\hat{R}}_{D}-R_{D}\right)R_{D}^{-1}\left({\hat{R}}_{D}-R_{D}\right)\right)\\ \;\;\;=\min_{R_{D}}\mathrm{Tr}\left({\hat{R}}_{D}R_{D}^{-1}{\hat{R}}_{D}\right)-2\mathrm{Tr}\left({\hat{R}}_{D}\right)+\mathrm{Tr}\left(R_{D}\right). \end{array}$$

where Tr(·) denotes the trace of a matrix. Since $R_{D}$ is a Toeplitz matrix, it can be written as T(u), where T(·) is the Toeplitz operator and u is a complex vector. Therefore, (19) can be further transformed into

(20) $$\begin{array}{c} \;\;\;\;\;\;\;\;\;\;\;\;\;\;\;\;\;\;\;\;\;\;\;\;\;\;\;\;\;\;\;\min_{R_{D}}\mathrm{Tr}\left({\hat{R}}_{D}R_{D}^{-1}{\hat{R}}_{D}\right)-2\mathrm{Tr}\left({\hat{R}}_{D}\right)+\mathrm{Tr}\left(R_{D}\right)\\ \;\;\;\;\;\;\;\;\;\;\;\;\;\;\;\;\;=\min_{u}\left(\mathrm{Tr}\left(W\right)-2\mathrm{Tr}\left({\hat{R}}_{D}\right)+\mathrm{Tr}\left(T\left(u\right)\right)\right)\\ s.t.W⪰{\hat{R}}_{D}T{\left(u\right)}^{-1}{\hat{R}}_{D},T\left(u\right)⪰0\\ \;\;\;\;\;\;\;\;\;\;\;\;\;\;\;\;\;=\min_{u}\left(\mathrm{Tr}\left(W\right)-2\mathrm{Tr}\left({\hat{R}}_{D}\right)+\mathrm{Tr}\left(T\left(u\right)\right)\right)\\ s.t.\left[\begin{array}{cc} W & {\hat{R}}_{D}\\ {\hat{R}}_{D} & T(u) \end{array}\right]⪰0,T\left(u\right)⪰0, \end{array}$$

where W is an auxiliary variable and ⪰ denotes the positive semidefinite matrix inequality.

Equation (20) successfully transforms the minimization term in (18) into a convex optimization problem. Next, we transform the three equality constraints in (18) into convex constraints.

Let the unquantized received-signal covariance matrix of the sparse array S be

(21) $$\begin{array}{cc} {\hat{R}}_{S} & =E\{X_{S}X_{S}^{H}\}\\ \; & =\frac{1}{N}X_{S}X_{S}^{H}. \end{array}$$

By using (8) and (9), we can establish the relationship between ${\hat{R}}_{S}$ and ${\hat{R}}_{D}$ as

(22) $${\hat{R}}_{S}=\Gamma {\hat{R}}_{D}\Gamma^{H}.$$

Since the optimization variables in (19) are u and the auxiliary matrix W, the term $-2\;\mathrm{Tr}({\hat{R}}_{D})$ in (19) is a data-dependent constant and can be dropped from the objective without affecting the optimum. After dropping this constant and applying the change of variable $R_{D}=T\left(u\right)$ together with (22), the objective is expressed purely in terms of quantities that appear in the final SDP:    

(23) $$\begin{array}{cc} \min_{R_{D}} & \|R_{D}^{-\frac{1}{2}}\left({\hat{R}}_{D}-R_{D}\right){\|}_{F}^{2}\\ ⟺ & \min_{u,W}\left(\mathrm{Tr}\left(W\right)+\mathrm{Tr}\left(T\left(u\right)\right)\right)\\ s.t. & \left[\begin{array}{cc} W & {\hat{R}}_{S}\\ {\hat{R}}_{S} & \Gamma T\left(u\right)\Gamma^{H} \end{array}\right]⪰0,T\left(u\right)⪰0, \end{array}$$

where the equivalence is up to a data-dependent constant. By substituting (23) and (21) into (18), the first equality constraint is transformed into a convex constraint, yielding

(24) $$\begin{array}{cc} \min_{u,W} & \left(\mathrm{Tr}(W)+\mathrm{Tr}(T(u))\right)\\ s.t. & \left[\begin{array}{cc} W & {\hat{R}}_{S}\\ {\hat{R}}_{S} & \Gamma T\left(u\right)\Gamma^{H} \end{array}\right]⪰0,T\left(u\right)⪰0,\\ \; & {\hat{R}}_{S}=\frac{1}{N}X_{S}X_{S}^{H},\\ \; & Y_{S}=Q\left(X_{S}\right). \end{array}$$

Next, the second equality constraint in (18) can be relaxed into a convex inequality constraint using the positive semidefiniteness of ${\hat{R}}_{S}$. First, the equality constraint is relaxed to

(25) $${\hat{R}}_{S}⪰\frac{1}{N}X_{S}X_{S}^{H}.$$

By utilizing the positive semidefinite property of ${\hat{R}}_{S}$, we have

(26) $$N{\hat{R}}_{S}⪰X_{S}X_{S}^{H},{\hat{R}}_{S}⪰0.$$

By the Schur complement [38], (26) is equivalent to

(27) $$\left[\begin{array}{cc} N{\hat{R}}_{S} & X_{S}\\ X_{S}^{H} & I \end{array}\right]⪰0,{\hat{R}}_{S}⪰0.$$

Inserting (27) into (24), we relax the second equality constraint into a convex constraint, yielding

(28) $$\begin{array}{cc} \min_{u,W,X_{S},{\hat{R}}_{S}} & \left(\mathrm{Tr}(W)+\mathrm{Tr}(T(u))\right)\\ s.t. & \left[\begin{array}{cc} W & {\hat{R}}_{S}\\ {\hat{R}}_{S} & \Gamma T\left(u\right)\Gamma^{H} \end{array}\right]⪰0,T\left(u\right)⪰0,\\ \; & \left[\begin{array}{cc} N{\hat{R}}_{S} & X_{S}\\ X_{S}^{H} & I \end{array}\right]⪰0,{\hat{R}}_{S}⪰0,\\ \; & Y_{S}=Q\left(X_{S}\right). \end{array}$$

Finally, the third equality constraint in (18) can be relaxed into a convex constraint using properties of the sign function. According to (21), the sign consistency of 1.5-bit quantization can be written as

(29) $$\begin{array}{cc} \; & Q\left(ℜ\left(X_{S}\right)\right)=ℜ\left(Y_{S}\right),\\ \; & Q\left(ℑ\left(X_{S}\right)\right)=ℑ\left(Y_{S}\right). \end{array}$$

Using (15), (29) can be further written as

(30) $$\begin{array}{cc} \; & \mathrm{sign}\left(ℜ\left(X_{S}\right)-\lambda \right)+\mathrm{sign}\left(ℜ\left(X_{S}\right)+\lambda \right)=2ℜ\left(Y_{S}\right),\\ \; & \mathrm{sign}\left(ℑ\left(X_{S}\right)-\lambda \right)+\mathrm{sign}\left(ℑ\left(X_{S}\right)+\lambda \right)=2ℑ\left(Y_{S}\right). \end{array}$$

Using the fact that the square of any real number is non-negative, we can relax (30) to

(31) $$\begin{array}{cc} \; & \mathrm{vec}\left(\mathrm{sign}\left(ℜ\left(X_{S}\right)-\lambda \right)+\mathrm{sign}\left(ℜ\left(X_{S}\right)+\lambda \right)\right)⊙\mathrm{vec}\left(ℜ\left(Y_{S}\right)\right)\geq 0,\\ \; & \mathrm{vec}\left(\mathrm{sign}\left(ℑ\left(X_{S}\right)-\lambda \right)+\mathrm{sign}\left(ℑ\left(X_{S}\right)+\lambda \right)\right)⊙\mathrm{vec}\left(ℑ\left(Y_{S}\right)\right)\geq 0. \end{array}$$

where ⊙ denotes the Hadamard product. Since the sign function is not convex, (31) is still not a convex constraint. However, inspection of (31) shows that the sign function can be replaced by the unquantized data without affecting the validity of the inequality. Thus, we can relax (31) to

(32) $$\begin{array}{c} \mathrm{vec}\left(ℜ\left(X_{S}\right)-\lambda \right)+\mathrm{vec}\left(ℜ\left(X_{S}\right)+\lambda \right))⊙\mathrm{vec}\left(ℜ\left(Y_{S}\right)\right)\geq 0\\ \;⟺\mathrm{vec}\left(ℜ\left(X_{S}\right)\right)⊙\mathrm{vec}\left(ℜ\left(Y_{S}\right)\right)\geq 0,\\ \mathrm{vec}\left(ℑ\left(X_{S}\right)-\lambda \right)+\mathrm{vec}\left(ℑ\left(X_{S}\right)+\lambda \right))⊙\mathrm{vec}\left(ℑ\left(Y_{S}\right)\right)\geq 0\\ \;⟺\mathrm{vec}\left(ℑ\left(X_{S}\right)\right)⊙\mathrm{vec}\left(ℑ\left(Y_{S}\right)\right)\geq 0, \end{array}$$

At this point, the third equality constraint in (18) has also been relaxed into a convex constraint.

**Remark on the relaxation.** We emphasize the status of (32): it is the only genuinely loose step in the derivation, whereas the Schur-complement rewriting of the Frobenius objective and of the sample-covariance equality either are equivalent or tighten to standard SDP relaxations already used in the sparse covariance-fitting literature [29,37]. Four observations are in order. (i) The relaxation in (32) discards, in the forward direction, the exact value of the quantization threshold λ: any $X_{S}$ whose real and imaginary parts share the sign of the corresponding entries of $Y_{S}$ is admissible, regardless of the magnitude of λ. (ii) Nevertheless, the threshold is not entirely erased from the problem: λ controls the zero pattern of $Y_{S}$ (the zero-triad corresponds to $|x_{m}\left(t\right)|\leq \lambda$), and entries of $X_{S}$ associated with zero entries of $Y_{S}$ are unconstrained by sign but are still shaped by the covariance-fitting term. (iii) The relaxation preserves phase information since the sign products are imposed separately on the real and imaginary parts in (32). (iv) A tight empirical indicator of the looseness of (32) is the gap, observed in Section 4, between 1.5B-SCE and the same covariance-fitting SDP applied to unquantized data (denoted UNQ-SCE): this gap upper-bounds the information lost by the sign relaxation in our formulation. A tight analytical bound, for instance a fundamental limit on the minimum MSE achievable by any convex relaxation that preserves sign consistency, is beyond the scope of this paper and is left as future work.

**Tightness of the relaxation.** Collecting the observations above, the three transformations used in the derivation have the following tightness status. (a) The Schur-complement rewriting of the Frobenius objective in (20) is an equivalent reformulation: its optimum in the auxiliary matrix is attained at $W^{⋆}={\hat{R}}_{D}T{\left(u\right)}^{-1}{\hat{R}}_{D}$, so no optimum is lost. (b) The PSD relaxation of the sample-covariance equality in (25)–(27) is tight at the optimum whenever the minimizing $X_{S}$ has rank at most *M*, which the trace term in the objective implicitly encourages; this is the same tightness condition documented for SPA-style structured covariance fitting in [29,37], and it holds in our setting whenever the covariance-fitting objective dominates the sign-constraint slack, which is the case in the snapshot regime considered in Section 4. (c) The sign-product inequality in (32) is the only genuinely loose step, in the sense that its feasible set strictly contains that of (30); the size of this gap is numerically bounded above by the 1.5B-SCE to UNQ-SCE curve distance as shown in the numerical results, which stays within 2–3 dB on the coprime array across the tested SNR and snapshot ranges. In particular, (a) and (b) together certify that the SDP in (33) is a valid convex surrogate of the covariance-fitting problem in (18) whose only information loss is attributable to (c), and this loss is empirically small.

By substituting (32) into (28), and recalling from (23) that the data-dependent constant has already been dropped from the objective, we obtain the final covariance matrix fitting optimization problem:

(33) $$\begin{array}{cc} \min_{u,W,X_{S},{\hat{R}}_{S}} & \left(\mathrm{Tr}(W)+\mathrm{Tr}(T(u))\right)\\ s.t. & \left[\begin{array}{cc} W & {\hat{R}}_{S}\\ {\hat{R}}_{S} & \Gamma T\left(u\right)\Gamma^{H} \end{array}\right]⪰0,T\left(u\right)⪰0,\\ \; & \left[\begin{array}{cc} N{\hat{R}}_{S} & X_{S}\\ X_{S}^{H} & I \end{array}\right]⪰0,{\hat{R}}_{S}⪰0,\\ \; & \mathrm{vec}\left(ℜ\left(X_{S}\right)\right)⊙\mathrm{vec}\left(ℜ\left(Y_{S}\right)\right)\geq 0,\\ \; & \mathrm{vec}\left(ℑ\left(X_{S}\right)\right)⊙\mathrm{vec}\left(ℑ\left(Y_{S}\right)\right)\geq 0. \end{array}$$

Equation (33) is a standard semidefinite programming (SDP) problem, which can be solved using SDP algorithms such as SDPT3 [39] to obtain u, and hence the estimate of the unquantized ULA covariance matrix $R_{D}=T\left(u\right)$. Finally, using subspace methods such as MUSIC or ESPRIT, the estimated DOAs of the source signals can be obtained from T(u). In summary, the steps of the proposed estimator are given in Algorithm 1.
**Algorithm 1:** DOA estimator based on 1.5-bit sparse covariance matrix estimation (1.5B-SCE)
    **Require:** 1.5-bit quantized received signal matrix $Y_{S}$, quantization threshold λ, number of snapshots *N*, array selection matrix Γ.
    **Ensure:** Estimated DOAs of source signals.
      1:Compute the sample covariance matrix ${\hat{R}}_{S}=\frac{1}{N}Y_{S}Y_{S}^{H}$.      2:Solve the SDP problem in (33) to obtain T(u).      3:Use subspace methods such as MUSIC or ESPRIT to estimate the DOAs from T(u).


### Computational Complexity

The SDP in (33) has three classes of optimization variables: the Toeplitz parameter $u\in C^{M_{D}}$ where $M_{D}=\omega_{M}+1$ is the aperture of the virtual ULA; the auxiliary Hermitian matrices $W,{\hat{R}}_{S}\in C^{M\times M}$; and the snapshot block $X_{S}\in C^{M\times N}$. The dominant real-variable count is $m_{v}=\Theta \left(MN+M^{2}+M_{D}\right)$, which simplifies to $m_{v}=\Theta \left(MN\right)$ in the snapshot-rich regime N≳M. The two LMI constraints involve Hermitian blocks of size 2M×2M and (M+N)×(M+N), respectively, and the Toeplitz PSD constraint T(u)⪰0 is of size $M_{D}\times M_{D}$; the largest LMI block therefore has dimension $L_{\max}=M+N$, and the sum of LMI block sizes is $L_{\Sigma }=3M+N+M_{D}$. A primal-dual interior-point SDP solver such as SDPT3 [39] requires $O(\sqrt{L_{\Sigma }}\log\left(1/\epsilon \right))$ Newton iterations to reach an ϵ-accurate solution, and each Newton iteration is dominated by Cholesky factorization of the largest LMI block at cost $O(L_{\max}^{3})$ together with the formation and solution of the Schur complement at cost $O(m_{v}^{2}L_{\max}^{2}+m_{v}^{3})$ [38]. In the snapshot-rich regime N≳M, the LMI Cholesky term $O\left(L_{\max}^{3}\right)=O\left({\left(M+N\right)}^{3}\right)$ dominates the per-iteration cost, so the overall worst-case complexity scales as $O({\left(M+N\right)}^{3.5}\log\left(1/\epsilon \right))$; in the very-large-snapshot regime where the Schur-complement cost dominates, the bound degrades to $O\left(m_{v}^{3}\sqrt{L_{\Sigma }}\log\left(1/\epsilon \right)\right)=O\left({\left(MN\right)}^{3}\sqrt{M+N}\log\left(1/\epsilon \right)\right)$. In contrast, 1.5B-MUSIC [20] forms an M×M sample covariance, applies the arcsine law to recover the augmented coarray covariance on the virtual ULA, and then performs an $M_{D}\times M_{D}$ eigendecomposition at cost $O(M_{D}^{3})$, so 1.5B-SCE trades higher computation for the structured regularization benefit documented in Section 4. Because the cost of (33) grows steeply with *N* through the $X_{S}$ block, for very long snapshots or very large arrays a first-order reformulation (for example an ADMM splitting in which $X_{S}$, ${\hat{R}}_{S}$, and u are updated alternately, or a Burer–Monteiro factorization of the LMIs) would be a more practical implementation path; we leave such a solver-level study to future work.

**Accuracy–complexity trade-off.** Combining the analytical bounds above with the empirical runtime reported later in the numerical results, the two methods occupy opposite ends of the accuracy–complexity spectrum. 1.5B-MUSIC incurs a per-trial cost of $O(M_{D}^{3})$—below 20 ms on all tested apertures—but is statistically inefficient due to coarray processing [22] and caps the number of resolvable sources at $M_{D}-2$ on coprime arrays because of the hole in the difference coarray. 1.5B-SCE incurs a per-trial cost of $O({\left(M+N\right)}^{3.5}\log\left(1/\epsilon \right))$—4 s to 18 s on the same apertures—but recovers the full $M_{D}-1$ degrees of freedom and reduces the MSE of DOA estimation by roughly 2–12 dB in the coprime/low-snapshot regime as the numerical results indicate. In short, 1.5B-SCE is preferable when per-snapshot accuracy is the limiting factor (for example, offline post-processing, batch covariance estimation, or low-snapshot scenarios in which coarray MUSIC loses statistical efficiency), whereas 1.5B-MUSIC remains attractive for real-time, high-aperture tracking where per-trial latency dominates the design budget.

## 4. Numerical Results

In this section, we present simulation results of the 1.5B-SCE method and validate the performance of the proposed algorithm. Unless otherwise specified, the sparse array used for simulation in this section consists of 6 elements arranged as follows:

$$\begin{array}{cc} S_{n} & =\{0,1,2,3,7,11\},\\ S_{c} & =\{0,2,3,4,6,9\}, \end{array}$$

where $S_{n}$ is a nested array and $S_{c}$ is a coprime array. The nested array $S_{n}$ is composed of two 3-element ULA subarrays, with $M_{1}=M_{2}=3$; the coprime array $S_{c}$ is composed of two ULA subarrays with $M_{1}=2$ and $M_{2}=3$. In the experiments, all source signals have unit energy, and the number of sources is assumed to be known. The noise at each array element is zero-mean complex Gaussian white noise and is independent and identically distributed across elements. The signal-to-noise ratio (SNR) is defined as

$$\mathrm{SNR}=10\log_{10}\left(\frac{p^{2}}{\sigma^{2}}\right),$$

where *p* and σ are the source and noise powers, respectively. After solving the SDP problem in (33), the root-MUSIC algorithm is used to obtain the estimated DOAs ${\hat{\bar{\theta }}}_{k}$. In this section, the mean squared error (MSE) is used to measure the accuracy of DOA estimation, defined as

(34) $$\mathrm{MSE}=\frac{1}{K}\sum_{k=1}^{K}{\left({\bar{\theta }}_{k}-{\hat{\bar{\theta }}}_{k}\right)}^{2},$$

where ${\bar{\theta }}_{k}$ is the true DOA of the *k*-th source.

All experiments in this section were run on a computer with MATLAB 2025b, the Windows 11 operating system, an Intel Core i7-14700K CPU (3.4 GHz), and 64 GB of memory. For solving the SDP, we use the SDPT3 algorithm from the CVX convex optimization toolbox for MATLAB. Since it was shown in [21] that, under 1.5-bit quantization, the DOA estimation performance obtained from the closed-form covariance recovery is similar to that obtained by directly applying MUSIC to the sample covariance matrix, we simplify the experimental setup by using MUSIC applied directly to the sample covariance matrix as the 1.5-bit comparison algorithm, denoted as 1.5B-MUSIC. Additionally, in some experiments we include MUSIC under unquantized sparse arrays as a reference for the performance upper bound, denoted as UNQ-MUSIC. To isolate the benefit of the structured covariance-fitting regularization from the benefit of using 1.5-bit over 1-bit quantization, we also report two additional baselines built from the same SDP kernel as (33) but without the 1.5-bit sign constraints: UNQ-SCE, which applies the covariance-fitting SDP directly to the unquantized sample covariance; and OB-SCE, which applies it to a 1-bit quantized sample covariance (obtained by setting λ=0). UNQ-SCE thus acts as an empirical upper bound on what the proposed covariance-fitting formulation can achieve in the absence of quantization loss, while OB-SCE isolates the genuine 1.5-bit-over-1-bit benefit at the same level of regularization.

We first verify the number of resolvable sources that 1.5B-SCE can handle with different sparse arrays. As mentioned earlier, 1.5B-SCE uses the output of the sparse array S to estimate the covariance matrix of the ULA D with the same degrees of freedom; hence, when 1.5B-SCE is applied to an SLA, the number of resolvable sources depends only on the aperture of S and is independent of the type of S.

**Figure 1:**
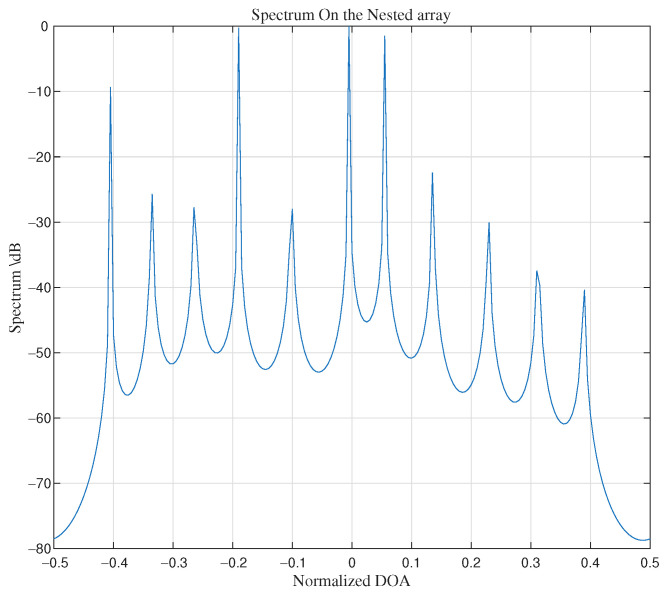
MUSIC spectrum after 1.5B-SCE recovers the covariance matrix on the nested array with 11 sources.

As shown in Figure 1, we plot the MUSIC spectrum after 1.5B-SCE recovers the covariance matrix on $S_{n}$, with SNR =10 dB, 100 snapshots, and 11 sources with different DOAs. From Figure 1, we observe 11 spectral peaks in total, which shows that for $S_{n}$ with 12 degrees of freedom, 1.5B-SCE can successfully resolve the maximum number of sources that it is theoretically able to resolve.

**Figure 2:**
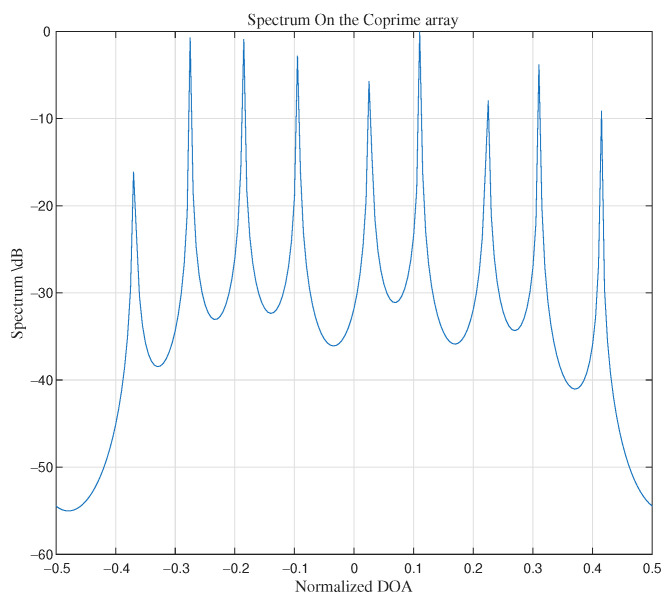
MUSIC spectrum after 1.5B-SCE recovers the covariance matrix on the coprime array with 9 sources.

As shown in Figure 2, we also plot the MUSIC spectrum after 1.5B-SCE recovers the covariance matrix on the coprime array, using the same SNR and number of snapshots as in Figure 1. However, since $S_{c}$ has 10 degrees of freedom, we use 9 sources with different DOAs. In Figure 2, there are 9 spectral peaks in total, indicating that 1.5B-SCE successfully resolves 9 sources. In contrast, 1.5B-MUSIC of [20] uses a difference co-array and discards the virtual elements outside the holes of the difference co-array of the coprime array, which reduces the effective degrees of freedom. For example, for the 6-element coprime array used in this paper, due to the hole at position 8, 1.5B-MUSIC can only exploit the difference co-array with 8 degrees of freedom, yielding a maximum of 7 resolvable sources. Therefore, compared with 1.5B-MUSIC, 1.5B-SCE increases the number of resolvable sources on coprime arrays.

In the following experiments, we examine the impact of the 1.5-bit quantization threshold λ on DOA estimation performance. The experiments are conducted at SNRs of 0 dB and 10 dB, with 100 snapshots and 5 sources with different DOAs. All results are averaged over 200 Monte Carlo trials. To better illustrate the performance of 1.5B-SCE, we also provide results for 1.5B-MUSIC and 1-bit MUSIC for comparison.

**Figure 3:**
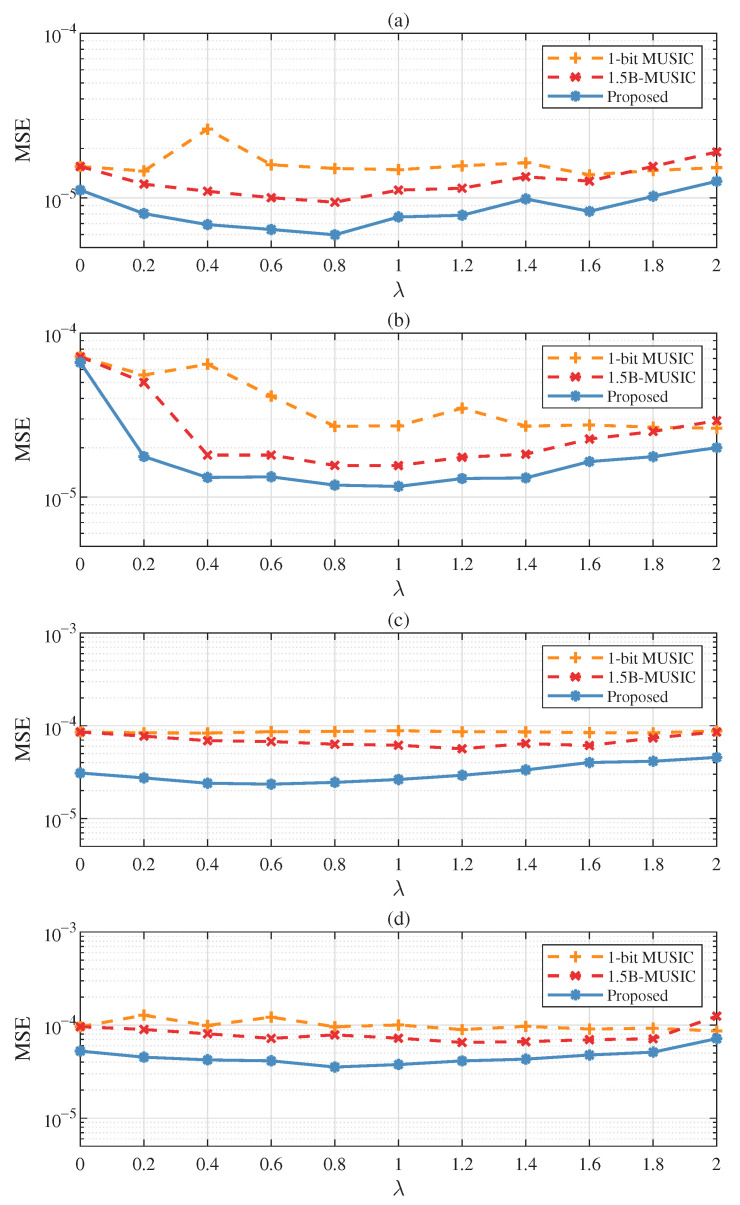
MSE of DOA estimation versus the quantization threshold λ under different SNRs. (**a**) Nested array at SNR =10 dB. (**b**) Nested array at SNR =0 dB. (**c**) Coprime array at SNR =10 dB. (**d**) Coprime array at SNR =0 dB.

As shown in Figure 3, 1.5B-SCE exhibits good DOA estimation performance under different quantization thresholds. In addition, 1.5B-SCE significantly outperforms 1.5B-MUSIC across different quantization thresholds, which validates the effectiveness of the proposed method. From Figure 3a,c, we observe that at SNR =10 dB, 1.5B-SCE achieves a lower MSE when the quantization threshold λ is between 0.4 and 1.0. However, when λ is too large, the performance of 1.5-bit quantization degrades to a level worse than that of 1-bit quantization. At SNR =10 dB, the best performance is achieved at λ=0.8, while at SNR =0 dB the best performance is achieved at λ=1.0. This indicates that the optimal value of λ is significantly related to the SNR. Furthermore, the performance gap between 1.5B-SCE and 1.5B-MUSIC remains relatively stable, indicating that 1.5B-SCE effectively suppresses finite-snapshot errors and improves DOA estimation performance. On the coprime array, as shown in Figure 3c,d, 1.5B-SCE also exhibits good DOA estimation performance, and its advantage over 1.5B-MUSIC remains relatively stable, validating the effectiveness of 1.5B-SCE on coprime arrays. Specifically, at SNR =10 dB, the best performance is achieved at λ=0.6; at SNR =0 dB, the best performance is achieved at λ=0.8. Comparing across arrays, at SNR =10 dB the performance gap between 1.5B-SCE and 1.5B-MUSIC is 2 dB on the nested array, while it is approximately 4 dB on the coprime array. At SNR =0 dB, the gap is 1.3 dB on the nested array and approximately 3.8 dB on the coprime array. This indicates that 1.5B-SCE offers a more significant performance improvement over 1.5B-MUSIC on coprime arrays. Finally, due to the sparsity of the nested array’s elements, its low-bit sampling exhibits noticeable fluctuations in numerical stability; however, this issue is beyond the scope of this paper.

**Practical guideline for selecting** λ**.** The numerical trends in Figure 3 also give rise to a short practical recipe for selecting the 1.5-bit quantization threshold. The best-performing λ tracks the signal-plus-noise scale $\sigma_{x}\approx \sqrt{P_{s}+\sigma^{2}}$, so $\lambda \in \left[0.6,1.0\right]\sigma_{x}$ is a reasonable operating range. In deployment, $\sigma_{x}$ can be estimated as ${\hat{\sigma }}_{x}\approx \sqrt{\frac{1}{M}\mathrm{tr}\left({\hat{R}}_{x}\right)}$ from a brief unquantized calibration burst if the front end allows one, or, when only 1.5-bit data are available, approximated from the average power of $Y_{S}$ together with an auxiliary noise-power estimate obtained from a signal-free interval. With ${\hat{\sigma }}_{x}$ in hand, we suggest the following three-item guideline:

(1) Set $\lambda =\alpha {\hat{\sigma }}_{x}$ with α∈[0.6,1.0].
(2) Choose α∈[0.6,0.8] when the operating SNR is high (≳10 dB) so that more samples exceed the threshold and the three-level quantizer preserves large-magnitude signal structure; choose α∈[0.8,1.0] when the operating SNR is low (≲0 dB), to avoid over-quantizing the noise floor into the non-zero levels.
(3) When the operating SNR is unknown or time-varying, α=0.8 is a robust default, since it lies inside the best-performing band on both the nested and coprime layouts of Figure 3 across the tested SNR range.

A closed-form or data-driven rule for adaptively selecting λ as a function of the noise level and the array geometry is an interesting direction for future work.

**Figure 4:**
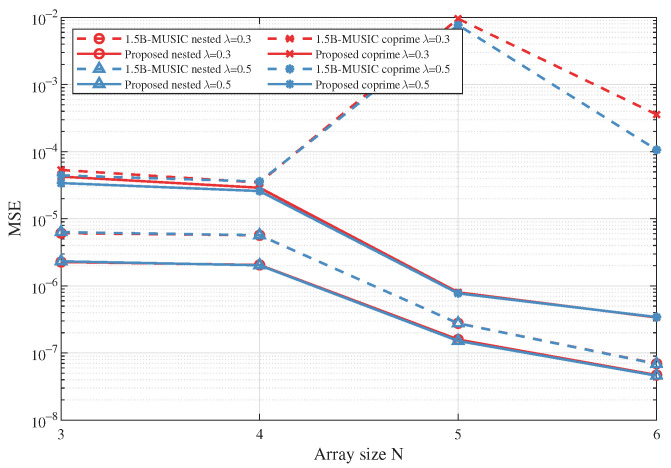
MSE of DOA estimation versus array size on coprime and nested arrays at SNR =10 dB and 100 snapshots.

We first examine how the proposed estimator scales with array size. We use both coprime and nested arrays with parameters $M_{1}=M_{2}$ chosen so that the total aperture grows, and report MSE versus the number of physical elements. The experiment is conducted at SNR =10 dB with 100 snapshots and K=3 sources. The results in Figure 4 show that, on both array geometries, the MSE of 1.5B-SCE decreases monotonically as the array size grows and stays consistently below that of 1.5B-MUSIC, and the relative ordering of the baselines is preserved. This indicates that the proposed covariance-fitting formulation scales gracefully to larger sparse arrays under both coprime and nested configurations.

**Figure 5:**
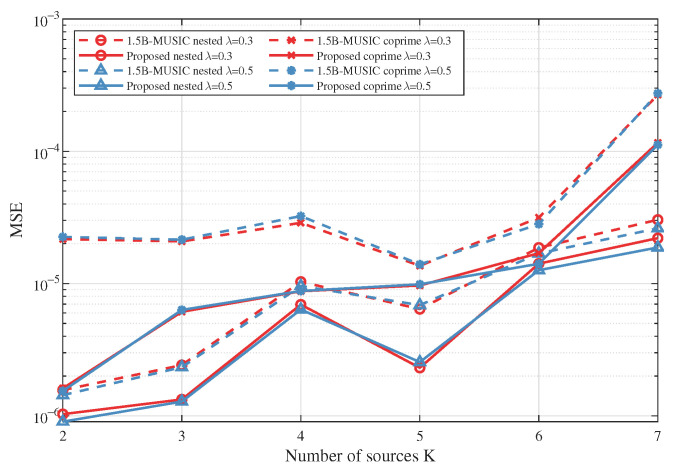
MSE of DOA estimation versus number of sources on nested and coprime arrays at SNR =10 dB and 100 snapshots.

Next, we verify the performance of 1.5B-SCE as the number of sources *K* varies, on both the nested array and the coprime array at SNR =10 dB with 100 snapshots. As shown in Figure 5, on both arrays 1.5B-SCE maintains an advantage over 1.5B-MUSIC across the range K∈{2,…,7}, and the degradation with increasing *K* is consistent with the reduced per-source effective aperture. For *K* approaching the degrees of freedom of the array, all estimators degrade, as expected.

We then verify the DOA estimation performance of 1.5B-SCE under different SNRs, ranging from −10 dB to 25 dB with a step size of 5 dB. The experiment uses 5 sources with different DOAs, 100 snapshots, and quantization thresholds λ=0.3 and λ=0.5. The reason is that at λ=0.5, 1.5B-SCE exhibits good DOA estimation performance on both nested and coprime arrays, while at λ=0.3, its performance is only average. For a fair comparison, we choose both thresholds in the experiments. Additionally, 1.5B-MUSIC and UNQ-MUSIC are included as comparison algorithms, together with the structured baselines UNQ-SCE and OB-SCE described above.

**Figure 6:**
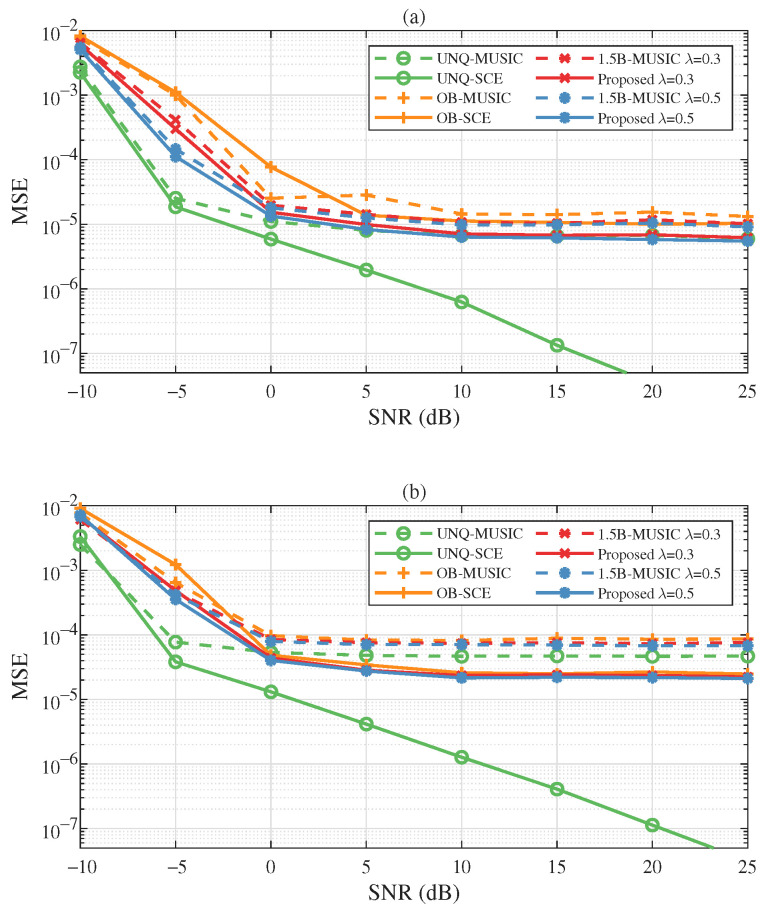
MSE of DOA estimation versus SNR under different quantization thresholds. The additional baselines UNQ-SCE and OB-SCE apply the same covariance-fitting SDP to the unquantized and 1-bit data, respectively. (**a**) Nested array. (**b**) Coprime array.

As shown in Figure 6, 1.5B-SCE exhibits good DOA estimation performance under different SNRs. In addition, 1.5B-SCE significantly outperforms 1.5B-MUSIC across different quantization thresholds, which validates the effectiveness of the proposed method. From Figure 6a, we observe that on the nested array, when λ=0.5 and SNR =20 dB, the estimation accuracy of 1.5B-SCE is about 2.3 dB better than that of 1.5B-MUSIC. Across different values of λ, its accuracy is close to that of UNQ-MUSIC, indicating that 1.5B-SCE effectively suppresses finite-snapshot errors and improves DOA estimation performance. From Figure 6b, we observe that on the coprime array, when λ=0.5 and SNR =20 dB, the estimation accuracy of 1.5B-SCE is about 10.5 dB better than that of 1.5B-MUSIC. When λ=0.3, it is about 5.1 dB better than 1.5B-MUSIC. In particular, at λ=0.5 and SNR =20 dB, the accuracy of 1.5B-SCE is about 9.0 dB better than that of UNQ-MUSIC, indicating that the proposed algorithm yields a more significant performance improvement on coprime arrays.

Three further observations follow once the structured baselines UNQ-SCE and OB-SCE are added to Figure 6. First, the gap between 1.5B-SCE and UNQ-SCE isolates the loss attributable to 1.5-bit quantization at the same level of structured regularization: on the coprime array this gap is typically 2–3 dB at moderate SNR, consistent with the 1.5-bit/unquantized loss reported for closed-form recovery in [21]. Second, the gap between 1.5B-SCE and OB-SCE isolates the genuine benefit of moving from 1-bit to 1.5-bit at fixed regularization: on the coprime array this gap reaches 3–5 dB in the high-SNR regime, because 1-bit quantization saturates while 1.5-bit retains coarse amplitude information. Third, the fact that 1.5B-SCE can match or exceed plain UNQ-MUSIC on the coprime array is consistent with the structured baselines: UNQ-SCE also outperforms UNQ-MUSIC on the coprime array, which shows that the advantage comes from the structured covariance-fitting regularization—not from the quantization itself. We therefore avoid interpreting the 1.5B-SCE/UNQ-MUSIC crossover as information gain from quantization.

Finally, we verify the DOA estimation performance of 1.5B-SCE under different numbers of snapshots, ranging from 20 to 196 with a step size of 16. The experiment uses 5 sources with different DOAs, SNR =10 dB, and quantization thresholds λ=0.3 and λ=0.5.

**Figure 7:**
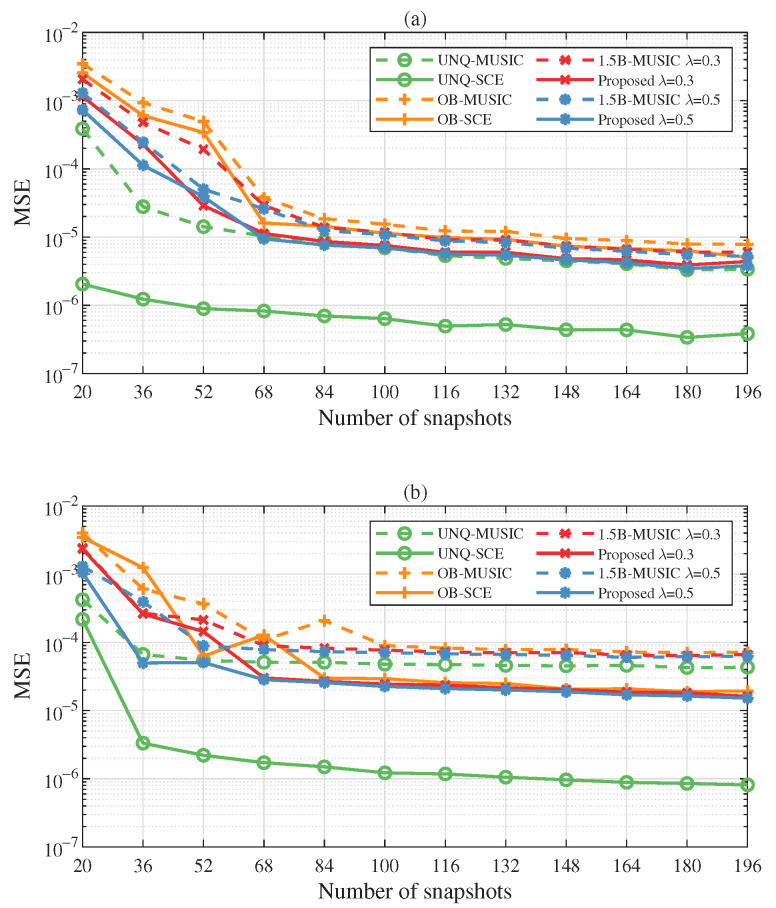
MSE of DOA estimation versus number of snapshots under different quantization thresholds. The additional baselines UNQ-SCE and OB-SCE apply the same covariance-fitting SDP to the unquantized and 1-bit data, respectively. (**a**) Nested array. (**b**) Coprime array.

As shown in Figure 7, 1.5B-SCE exhibits good DOA estimation performance across different numbers of snapshots. In particular, on the coprime array, 1.5B-SCE significantly outperforms UNQ-MUSIC across different quantization thresholds, which validates the effectiveness of the proposed method. When the number of snapshots is only 68, 1.5B-SCE achieves an estimation accuracy approximately 8.1 dB better than UNQ-MUSIC and approximately 10.1 dB better than 1.5B-MUSIC at λ=0.5. When the number of snapshots is 180, its performance is approximately 11.3 dB better than UNQ-MUSIC and approximately 12.5 dB better than 1.5B-MUSIC. However, as shown in Figure 7a, on the nested array the performance advantage of 1.5B-SCE is relatively modest, about 1.7 dB. Therefore, the proposed algorithm is more suitable for coprime arrays than for nested arrays. The same three-way reading that we applied to Figure 6 extends here: the 1.5B-SCE/UNQ-SCE gap quantifies the 1.5-bit loss at fixed regularization, the 1.5B-SCE/OB-SCE gap quantifies the genuine 1.5-bit-over-1-bit gain, and the 1.5B-SCE/UNQ-MUSIC crossover on the coprime array is matched by the UNQ-SCE/UNQ-MUSIC crossover, confirming that the advantage comes from the structured covariance-fitting regularization rather than from quantization.

**Figure 8:**
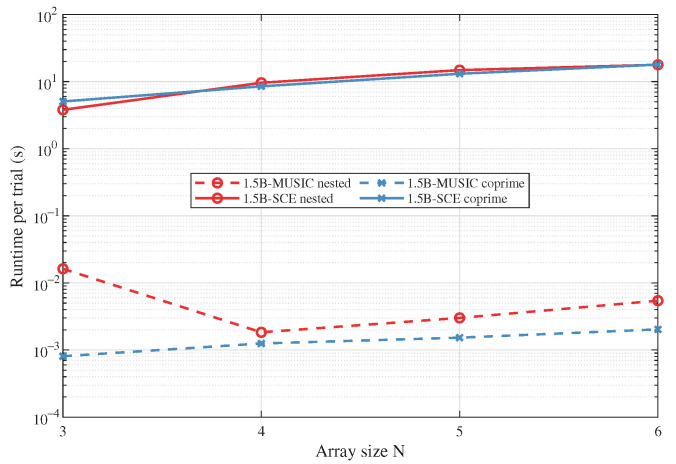
Average runtime per Monte Carlo trial of 1.5B-SCE and 1.5B-MUSIC versus array size on coprime and nested arrays at SNR =10 dB and 100 snapshots.

To complement the analytical complexity bounds derived in Section 3, we also report the empirical wall-clock runtime of 1.5B-SCE and 1.5B-MUSIC under the same array-size sweep as in Figure 4. Both methods are run in MATLAB R2025b on the same workstation; 1.5B-SCE uses CVX with the SDPT3 solver, and 1.5B-MUSIC consists of arcsine-law correction on the coarray followed by a root-MUSIC eigendecomposition. The reported value is the average of 20 independent Monte Carlo trials at SNR =10 dB and 100 snapshots. As shown in Figure 8, the runtime of 1.5B-MUSIC remains below 20 ms across all tested apertures, while that of 1.5B-SCE grows from about 4 s at M=6 to about 18 s at M=12 (virtual ULA aperture $M_{D}\;\leq \;42$); the gap of roughly three orders of magnitude is consistent with the asymptotic comparison $O({\left(M+N\right)}^{3.5}\log\left(1/\epsilon \right))$ versus $O(M_{D}^{3})$ in Section 3, and confirms that 1.5B-SCE trades wall-clock time for the structured-regularization gain documented above.

## 5. Conclusions

In this paper, we proposed a 1.5-bit sparse-array DOA estimation method for massive MIMO systems based on covariance matrix estimation, referred to as 1.5B-SCE. The method exploits the sign consistency between 1.5-bit quantized and unquantized signals, as well as the Toeplitz structure of the covariance matrix of ULAs, to transform the DOA estimation problem into a constrained optimization problem. The optimization problem is then relaxed into a convex problem that can be solved via semidefinite programming. Numerical results show that the proposed method effectively improves the DOA estimation performance of 1.5-bit sparse arrays; in particular, on coprime arrays in the low-snapshot regime, it approaches structured covariance-fitting baselines applied to unquantized data while clearly outperforming 1.5B-MUSIC and 1-bit covariance-fitting baselines. Several directions remain open, including data-driven or closed-form rules for selecting the quantization threshold λ, first-order SDP solvers (e.g., ADMM or Burer–Monteiro schemes) to reduce the per-iteration complexity, and extensions of the formulation to model-mismatch scenarios such as off-grid sources, mutual coupling, and gain/phase uncertainty.

## Figures and Tables

**Table 1:** Notation used in Section 3.

Symbol	Role	Description
$X_{S}\in C^{M\times N}$	latent (unknown)	unquantized signal matrix on sparse array S
$Y_{S}\in {\left\{-1,0,+1\right\}}_{C}^{M\times N}$	observed	1.5-bit quantized signal matrix
${\hat{R}}_{S}$	optimization variable	unquantized sample covariance on S
${\hat{R}}_{D}$	derived	sample covariance on virtual ULA D
$R_{D}=T\left(u\right)$	modeling target	Toeplitz ULA covariance, parameter u
W	auxiliary	Schur-complement slack matrix
Γ	known	selection matrix D→S, $\Gamma \in {\left\{0,1\right\}}^{M\times M_{D}}$
λ	hyper-parameter	1.5-bit quantization threshold

## Data Availability

The raw data supporting the conclusions of this article will be made available by the authors on request.
